# Beyond the Bench: Flagging Environmental Health Awareness on Beaches

**DOI:** 10.1289/ehp.114-a468

**Published:** 2006-08

**Authors:** Tanya Tillett

During the summer months, folks flock to the beach to enjoy the combined pleasures of sun and sea. Smart beachgoers know that before they take a dip, they should check whether any warning flags are flying, indicating hazardous conditions such as rip currents or the presence of jellyfish. Now, Galveston swimmers can look for a new “environmental alert” flag. The new flag warns beachgoers of air and weather conditions that could pose a health threat, especially to particularly vulnerable populations such as asthmatics, the elderly, and people with heart or lung disease.

The new flag reflects the translation of research findings into concrete community health education by investigators in the Asthma Pathogenesis Core of the University of Texas Medical Branch (UTMB) and codirectors of the Asthma Community Outreach and Education Core (COEC), in partnership with the Galveston Sheriff’s Office Beach Patrol, the Galveston Park Board of Trustees, and the Texas Commission on Environmental Quality.

Three years ago, investigators Sharon A. Petronella and Edward G. Brooks initiated the Gulf Coast Study of Urban Air Quality and Respiratory Function (GC SURF) to study pulmonary function in a cohort of lifeguards in Galveston. During the summers of 2003 through 2005 they collected pulmonary effects data on the GC SURF cohort by using portable spirometers, which measure the amount of inhaled and exhaled air. This allowed them to evaluate exposure to and effects of air pollutants and weather conditions including nitric oxide, nitrogen dioxide, ozone, particulate matter, wind speed, outdoor temperature, relative humidity, and solar radiation.

The data gathered helped the investigators determine particular times of day when changes in air quality could affect breathing health. Now, whenever the Texas Commission on Environmental Quality deems that air quality conditions exist that might affect vulnerable populations, the city’s 26 lifeguard towers and 7 free-standing beach stations deploy an orange flag and display information on posters and in brochures describing the particular environmental issues and guidelines for protecting health.

The program, which the COEC believes to be the first in the nation to enlist lifeguard participation in an environmental health public warning system, provides educational materials on ozone, fine particulates, and, as needed, red tide. The group has now also developed a partnership with the Galveston County Health District to display the environmental flags at each tower when water quality is less than optimal.

Petronella says the alert program is the result of a true collaborative effort between the partner organizations and is a solid indication of what can happen when a community comes together. The developers, who hope the orange flag alert program can be used as a model for other beaches, presented it at the annual meeting of the U.S. Lifesaving Association in Galveston in 2004.

In addition to the GC SURF flag alert program, the Asthma Pathogenesis Core of the UTMB COEC is also involved in other projects that focus on building connections between research, education, and community health. One of these, the Texas Emergency Department Asthma Surveillance Project, is a collaborative effort coordinated by Charles Macias of Baylor College of Medicine that links the databases of Baylor and three other Texas hospitals to track asthma-related emergency room visits. The results will aid in the development of an educational intervention program. Another project, Communities Organized Against Asthma and Lead, is an environmental justice consortium combining the educational outreach efforts of the COEC with community social services and health care providers. COEC investigators are also involved in a school asthma surveillance project.

“As researchers involved in the UTMB NIEHS Center Asthma Pathogenesis Core, we work in and with our community to identify problems and potential solutions related to our environment,” says Petronella. “Our COEC, however, allows us to take our work one crucial step further––by actually assisting the community with education, intervention, and development of policies that will effect positive change in the health of our residents and all visitors to our part of the Gulf Coast.”

In essence, Petronella says, the COEC forms the bridge from basic science to the public. “This is essential to our success,” she adds, “since the key to any public health research program is the use to which the data are put.”

## Figures and Tables

**Figure:**
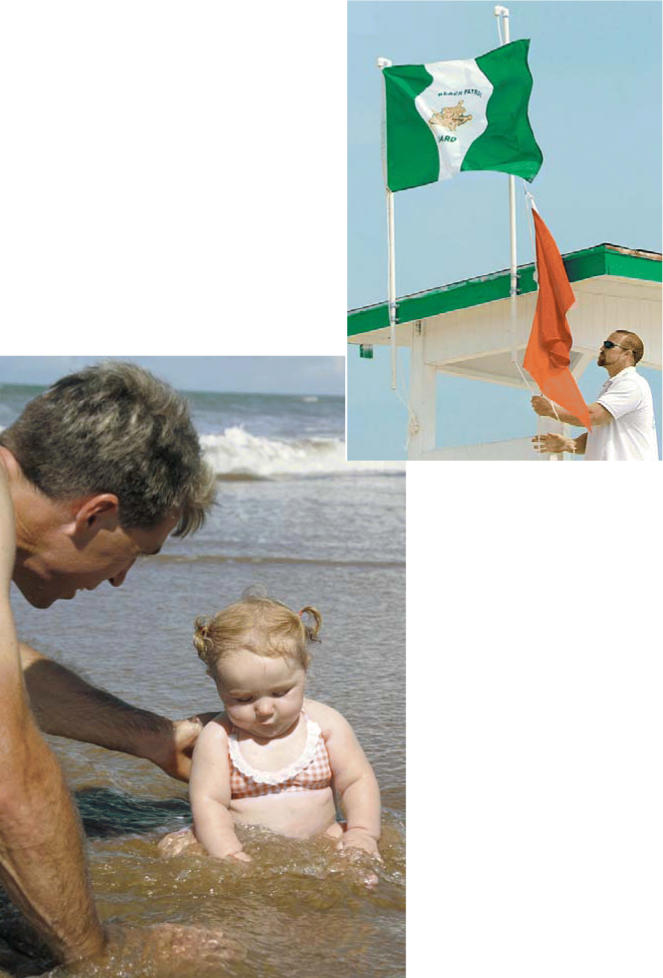
For better beachgoing A new orange flag indicates when Galveston beaches are experiencing poor air quality conditions that might affect vulnerable populations.

